# Does The Learning Curve Affect the Surgical, Functional, and Oncologic Outcomes in Bilateral Nerve-Sparing Robot Assisted Laparoscopic Prostatectomy?

**DOI:** 10.7759/cureus.5274

**Published:** 2019-07-30

**Authors:** Murat Ucar, Alkim T Varol, Kemal H Gülkesen, Ahmet E Caylan, Ömer Kutlu, Erol Güntekin

**Affiliations:** 1 Urology, Akdeniz University Faculty of Medicine., Antalya, TUR; 2 Urology, Akdeniz University Faculty of Medicine, Antalya, TUR; 3 Biostatistics and Medical Informatics, Akdeniz University Faculty of Medicine, Antalya, TUR

**Keywords:** robot-assisted prostatectomy, prostate cancer, da vinci robotic surgical system, urinary continence, potency

## Abstract

Introduction

Nowadays, the expectations for functional prostatectomy outcomes are quite high. Robot-assisted laparoscopic radical prostatectomy (RALRP) has become an increasingly common treatment option for men with localized prostate cancer. In this study, we aimed to present the results of our bilateral nerve-sparing RALRP procedure and to evaluate the effects of the learning curve (LC) on perioperative data, early oncologic, and functional outcomes.

Methods

The records of 132 RALRP cases performed between January 2016 and March 2019 by a single surgeon experienced in open and laparoscopic radical prostatectomy were evaluated retrospectively. Results of 91 cases with the bilateral nerve-sparing technique were analyzed. The learning curve was determined using the moving average method. LC analysis using the moving average method showed that the LC stabilized between cases 40 and 50. So, patients were divided into two groups: group 1 consisted of the first 45 cases, while group 2 consisted of 46-91^st^ cases. The groups were compared in terms of surgical, functional, and oncologic outcomes.

Results

The mean duration of surgery was significantly reduced in the second group (250 vs 235 min, p <0.002). However, there was no statistically significant difference between the groups in terms of hemoglobin decrease, hospitalization and catheterization time, and intraoperative and postoperative complication rates. The rates of pT2 cancers’ positive surgical margins (PSMs) were 32.4% and 19.4%, respectively. The recovery rate of continence in all the patients was 90.1% at 12 months. The potency ratios were calculated as 33.8% at 12 months. There was no statistically significant difference between the groups in terms of potency and continence rates at 3 months and 12 months, postoperatively.

Conclusion

For surgeons experienced in retropubic radical prostatectomy (RRP) and laparoscopic radical prostatectomy (LRP) surgeries, RALRP is a safe and feasible surgical procedure for both oncological and functional outcomes even during the learning curve.

## Introduction

Prostate cancer is the second most frequent malignancy (after lung cancer) in men worldwide, counting 1,276,106 new cases (7.1%) and causing 358,989 deaths (3.8% of all deaths caused by cancer in men) in 2018 [1]. According to the cancer statistics collected in Turkey, prostate cancer is the second most common type of cancer in men of all ages with the incidence of 11.8% [2]. Radical prostatectomy (RP) is an accepted and approved treatment for localized prostate cancer (PCa) in patients with a life expectancy of at least 10 years [3]. In order to reduce the morbidity of retropubic RP in the surgical treatment of PCa, Schuessler et al. developed laparoscopic radical prostatectomy in 1992 and later in 2000, Binder et al. presented a robot-assisted radical prostatectomy technique [4-5]. RALRP provides the benefits of minimally invasive surgery but has the potential to shorten the long learning curve observed with standard LRP, which is thought to be approximately 50 procedures [6-7]. In addition, although RALRP has disadvantages such as increased cost and inability to detect tissue or suture tension due to insufficient tactile sensation, it offers significant technical advantages over LRP including magnified three-dimensional images, increased tool maneuverability, and minimization of tremors [6-8]. While achieving the desired oncological results, no matter which technique is applied, it is not possible to avoid functional complications that affect social life after RP. Unfortunately, functional complications may affect patients to the extent that they overlook the oncologic control.

Erectile dysfunction is one of the most common functional complications after RP [8]. In order to prevent this complication, the bilateral or unilateral nerve-sparing technique can be applied to the patients who were found suitable during the preoperative evaluation. Such neuroprotective surgery is accepted as a technically demanding procedure. In this study, we retrospectively evaluated the outcomes of bilateral nerve-sparing RALRP cases performed by a single surgeon (ÖK) in our clinic and investigated whether the learning curve affected surgical, oncologic, and functional outcomes.

## Materials and methods

Patient selection

The data obtained from patients who underwent RALRP in our clinic were retrospectively evaluated. A single surgeon (ÖK), who is highly experienced in open and laparoscopic surgery, has performed all operations since January 2016. During this time, more than 150 robotic surgical procedures were performed and 132 of them were RALRP procedures. Between January 2016 and March 2019, there were 132 consecutive RALRP operations performed as a treatment of clinically localized prostate cancer. Ninety-one consecutive cases that underwent bilateral nerve-sparing procedure were included in this study. The first 45 cases were assigned to group 1 and the rest of the cases were assigned to group 2. The groups were similar in terms of preoperative clinical features (Table 1).

**Table 1:** Preoperative demographic characteristics

		Group 1 (1-45)	Group 2 (46-91)	p
Age, mean±SD		63.9±5.6	63.1±7.0	0.526
PSA, median (25th-75th percentile)		6.0 (4.7-8.0)	6.6 (4.9-9.4)	0.205
Biopsy Gleason score, n (%)	6	36 (80.0)	30 (65.2)	0.114
	7-8	9 (20.0)	16 (34.8)	
D’amico Risk group, n (%)	Low	30 (66.7)	22 (51.2)	0.309
	Intermediate	9 (20.0)	14 (32.6)	
	High	6 (13.3)	7 (16.3)	

Surgical technique

All RALRP procedures were performed by the transperitoneal approach. The surgery was performed by using a 4-arm Da Vinci Xi HD Surgical System (Intuitive Surgical Inc., Sunnyvale, CA, USA) with 6 trocar ports. As previously described by Zorn et al. [9], the procedure was started by first finding and ligating vas deferens and then dissection of seminal vesicles. Then, by turning to the front of the prostate, the dorsal vein complex was ligated and cut. The neurovascular bundle (NVB) was completely released and the prostate was dissected from the bladder neck. All procedures were done with the intent of bilateral full or partial nerve-sparing dissection involving a high anterior release of the neurovascular bundles. Nerve-sparing procedure was performed using a clips technique without the use of monopolar or bipolar cautery. Two 15-cm 3-0 V-lock stitches (3-0, 17 mm, ½) are usually used to perform the urethrovesical anastomosis. At the end of the surgery, an 18Fr Foley catheter was inserted. The catheter was inflated with 10 ml of saline. Pelvic lymph node dissection (PLND) was routinely performed in patients with Gleason score ≥ 4+3 or prostate-specific antigen (PSA) ≥ 10 ng/ml. The nerve-sparing procedure was not performed in patients that had preoperative International Erectile Function Index-5 (IIEF-5) score ≤17, suspected strong extracapsular spread on MRI or PSA levels above ≥10 ng/dl. Perioperative parameters such as duration of surgery, whether PLND or NSP were performed, and intraoperative complications were recorded. Postoperative parameters including hematocrit change, length of hospital stay, and time of catheter removal were also noted.

Complications

The modified Clavien Classification System was used to stratify all complications [10]. All complications that occurred within 30 days postoperatively were included.

Pathologic analysis

Uropathology clinicians in our institution evaluated all samples. Positive surgical margin (PSM) was defined as a tumor present at the inked margin. Location of PSM was noted. Patients with the extension of the tumor through the prostatic capsule were considered to have extracapsular-extension (pT3).

Follow-up

Since the RALRP is a transperitoneal procedure, each patient underwent cystography before catheter removal due to the possibility of urinary leakage causing peritonitis. The catheters were removed in patients whose cystography did not reveal any urine extravasation. In the majority of patients, the catheter was removed on the 7th postoperative day. The first measurement of serum prostate-specific antigen (PSA) was done 4-6 weeks after surgery. Biochemical relapse was defined as a two PSA measurement of above 0.2 ng/mL. Potency was defined as the ability to achieve a sufficient erection for penetration with or without the use of a phosphodiesterase inhibitor and was evaluated by patient interviews at 3 and 12 months postoperatively. Full urine continence was defined as no urinary incontinence and no use of any incontinence products and was evaluated at the postoperative 3rd and 12th months.

Data analysis

The patients’ medical records were analyzed retrospectively. Preoperative and perioperative data, as well as tumor features, were recorded. The operating time was defined as “skin to skin” time. The 2002 tumor, node, metastasis (TNM) staging system was used for clinical and pathological staging. Postoperative functional continence results and potency rates were evaluated by one-to-one or telephone interviews.

Statistical analysis

The SPSS 22.0 software program was used for statistical analysis. The normality was tested by using Shapiro-Wilk test. Groups were compared using t-test or Mann- Whitney U test. Nominal data were compared by Chi-square tests. Alpha significance level less than 0.05 was considered to be statistically significant.

## Results

**Figure 1:**
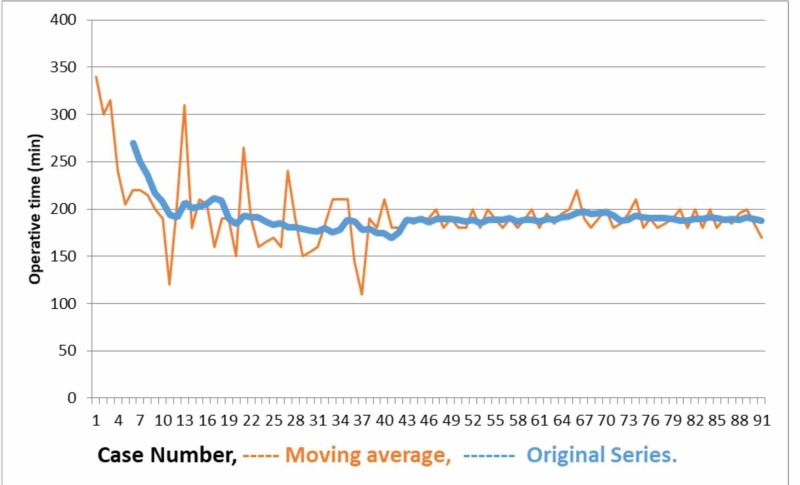
Time taken to perform robot-assisted laparoscopic radical prostatectomy (RALRP) in each case. Moving average curve of RALRP.

The patients were divided into two groups as group 1 (1st-45th cases) and group 2 (46th-91st cases). Preoperative clinical characteristics of both groups were similar (Table 1). The median age of the entire cohort was 63.4 years (mean 40-77) and the median PSA was 7.7 ng/ml. No statistically significant difference was found between the two groups in terms of D'amico risk classification and biopsy Gleason scores (p> 0.05). The moving average method was used to analyze the learning curve and as shown in Figure 1, it stabilized between 40th and 50th cases.

Based on the intraoperative data, none of the patients required converting to the open surgery. The evaluation of the operation time showed that although it was above average for the first cases in group 1, the learning curve rapidly plateaued. The operation time was significantly lower in group 2 (250 min in group 1 vs 235 min in group 2, p <0.002). The mean hospital stay was 8 days and was similar in both groups. The median time for Foley catheter removal was 7 days in both groups. Only one patient developed urinary retention after the Foley catheter was removed and a urethral catheter was required. The groups’ detailed perioperative and postoperative data are presented in Table 2.

**Table 2:** Perioperative and postoperative data

	Group 1 (1-45)	Group 2 (46-91)	p
Operation time, median (25th-75th percentile)	250 (227.5-270)	235 (225-241.3)	0.002
Duration of hospitalisation, median (25th-75th percentile)	8.0 (7.0-8.0)	8.0 (7.8-8.0)	0.674
Duration of cathateterization, median (25th-75th percentile)	7.0 (7.0-7.0)	7.0 (7.0-7.0)	
Hemoglobin decrease, median (25th-75th percentile)	1.9 (1.2-2.4)	2.1 (1.0-2.5)	0.631
Postoperative RBC Transfusions (%)	3 (6.6)	3 (6.5)	0.334
Mean Follow-up (months)	23.0 (20.5-29.0)	20.0 (16.0-22.0)	<0.001

Pathological and clinical follow-up results are shown in Table 3. The PSM rates were found to be 35.6% for group 1 and 26.1% for group 2 (p = 0.328). When PSM for pathological stage T2 was evaluated separately, it was observed that the PSM rate decreased clinically, although it was not statistically significant at 32.4% and 19.4% for the groups 1 and 2, respectively. In addition, extracapsular invasion (ECI) was detected in both groups (17.8% and 19.6%, respectively), but again there was no significant difference between the groups (p = 0.827). None of the patients had lymphovascular invasion. There was no significant difference between the two groups in terms of pathological Gleason score, perineural, seminal vesicle invasion, and pathological T stage (p> 0.05).

**Table 3:** Comparison of clinical and pathological outcomes

		Group 1 (1-45)	Group 2 (46-91)	p
Surgical margin status, n (%)	Negative	29 (64.4)	34 (73.9)	0.328
	Positive	16 (35.6)	12 (26.1)	
Surgical margin status, n (%) in pT2	Negative	25 (67.5)	29 (80.5)	0.206
	Positive	12 (32.4)	7 (19.4)	
Extracapsular invasion, n (%)	Yes	8 (17.8)	9 (19.6)	0.827
	No	37 (82.2)	37 (80.4)	
Lymphovascular invasion, n (%)	Yes	0 (0.0)	0 (0.0)	-
	No	45 (100.0)	46 (100.0)	
Perineural invasion, n (%)	Yes	37 (82.2)	41 (89.1)	0.346
	No	8 (17.8)	5 (10.9)	
Seminal vesicle invasion, n (%)	Yes	3 (6.7)	4 (10,9)	0.479
	No	5 (10.9)	41 (89.1)	
Pathological Gleason score, n (%)	6	37 (82.2)	29 (63.0)	0.040
	7-8	8 (17.8)	17 (37.0)	
Lymph node positive, n (%)	Yes	0 (0.0)	0 (0.0)	-
	No	45 (100.0)	46 (100.0)	
Pathological stage, n (%)	T2	37 (82.2)	36 (78.3)	0.635
Biochemical recurrence	Yes	0	0	
	No	34	29	

The mean follow-up times for groups 1 and 2 were 23 and 20 months, respectively. Functional results with evaluations of potency and continence of patients that had complete follow-ups in the postoperative period are presented in Table 4.

**Table 4:** Functional outcomes

	Group 1; n (%)	Group 2; n (%)	p
Continence			
3 months	15/38 (39.5%)	13/33 (39.4)	0.995
12 months	35/38 (92.1%)	29/33 (87.9%)	0.697
Potency			
3 months	2/38 (5.3%)	2/33 (6.1%)	>0.999
12 months	15/38 (39.5%)	9/33 (27.3%)	0.278

None of the patients required blood transfusion throughout the operation, but during the postoperative hospitalization period, hemoglobin decrease was detected in three patients from each groups, requiring a blood transfusion. Hemoglobin decrease was 1.9 g/dl and 2.1 g/dl in groups 1 and 2, respectively (p = 0.631). The majority of the complications in both groups were Clavien Grade 1 and 2 complications (75% vs 71%, respectively). One patient in group 1 had bladder perforation that was diagnosed and repaired perioperatively during the same session. This patient remained catheterized for a long time in the postoperative period and as a result developed urethral stricture, which was treated endoscopically in a different session. The patient has no voiding problem at this time. Another patient developed prolonged ileus, which was managed with intravenous fluids and temporary restriction of oral intake that resulted in the resolution of the symptoms. Clavien Gr 4a complication was detected in only one patient. This patient developed pulmonary thromboembolism and was treated medically with no pulmonary function limitation reported after the treatment was completed. The number and details of complications are presented in Table 5.

**Table 5:** Surgical complications stratified by the modified Clavien Classification System

Clavien system	Complications	Group 1	Group 2	Management
Overall		8	7	
I	Urinary retention	1		Prolonged catheter duration
I	Anastomosis site leakage	2	2	Prolonged catheter duration
II	Hb decline	3	3	Blood transfusion
IIIa	Subileus		1	Medical treatment
IIIb	Ureteral stricture	1		Internal urethrotomy
IIIb	Bladder perforation	1		Primer bladder repair
IVa	PTE		1	Medical treatment
PTE: Pulmonary thromboembolism				

## Discussion

RP is accepted as a first-line treatment modality for patients with organ-confined PCa and life expectancy of more than 10 years [3]. In this group of patients, who usually do not have any signs and symptoms other than elevated blood PSA values, attention is focused on both the oncologic and functional outcomes. Surgical technique, as well as the surgeon's experience, are crucial factors in achieving the desired oncological and functional results. Bianco et al. [11] suggested that cancer control, continence, and potency, also known as a trifecta, could be used as variables in evaluating the outcome of the surgery. Nowadays, with the widespread use of minimally invasive surgery, it has been advocated that adding postoperative complications and negative surgical margin criteria to the trifecta, making it a pentafecta, will be more realistic in meeting the patients’ expectations [12].

 Binder and Kramer [5] performed the first RALRP procedure in May 2000 and since then it has become a widely accepted treatment for PCa all over the world. RALRP provides the surgeon with high-resolution enlargeable three-dimensional images, increased maneuverability of the instruments used, and high light power [6]. However, as with any new surgical technique, RALRP has a learning curve. There is no consensus on the optimal way to determine the learning curve of a surgical procedure, but traditionally, the duration of surgery has been widely used to assess it. Zorn et al. suggested that completion of 120 RALRP procedures was needed to achieve a skin-to-skin operation time of less than 4 hours [13]. However, Islamoglu et al. [14] used the moving average method to find the cut-off point in determining the learning curve in RALRP, and found that at least 50 RALRP cases were required to gain competence, even for a surgeon highly experienced in LRP. They also emphasized that faster determination of trocar positions and placement and reduction of docking time had a positive effect on surgical technique as well as operation time [14]. Plossourd et al. [15] emphasized that the previous experience in laparoscopy was important for RALRP. Many centers have reported switching from open surgery to robotic surgery without prior laparoscopic training, but it was noted that the RALRP procedures performed after gaining laparoscopic experience resulted in shorter operation times and gave additional value to their work [6,14-16].

There are two crucial aspects of the RALRP learning curve. First, to perform the procedure safely and with clear margins, and second, to perform a nerve-sparing procedure. Menon et al. [6], Ahlering et al. [16], and Artibani et al. [17] reported similar complication rates at the end of 20 open prostatectomy and 100 LRP cases. The same studies reported that the learning curve in RALRP was shorter, at least in terms of complications. In our study, the RALRP learning curve for an experienced surgeon who performed more than 100 RRP and LRP procedures was found to have stabilized between 40 and 50 cases. Based on our results, we believe that this number is sufficient for the completion of the learning curve in RALRP and adaptation of the surgical team to the process.

Potency is one of the most difficult functional outcomes to compare after RP. Apart from the surgeon or technical approach, many factors, including the age of the patient, type and quality of nerve protection, and the use of medications have a significant impact on potential recovery. Current potency assessments are done with unconfirmed surveys and open interviews, but there is a need for a standardized potency assessment.

The potency rate has been reported to be between 31% and 86% in patients who underwent bilateral nerve-sparing RRP surgery and followed for a minimum of 12 months [18-19]. In a series that evaluated robotic surgeries performed between 2010 and 2015, the reported potency rates ranged from 30% to 90% [20-22]. Another study evaluated the effects of surgical experience on RALRP results between 1-300th cases, 301-500th cases, and 501-700th cases and reported that although surgical time, blood loss, surgical margin positivity rate in pT2 tumors, and the rate of continence without pad use have improved with surgical experience, there was no difference in the potency rates [23]. Zorn et al. [9] investigated the effects of the learning curve at the postoperative 3, 6 and 12 months and reported that the mean percentage of return to basal sexual function was similar for all groups (1st group 1-50th cases, 2nd group 51-100th, and 3rd group 101-150th cases). Similarly, in our study, there was no significant difference in the potency outcomes between the groups. The mean potency rate in our study was 33.8%. Although this rate is consistent with the literature, we believe that it does not accurately reflect our actual potency rates. We believe that potency rates might have been much higher if all our patients had access to PDE5 inhibitors in the postoperative period, which was not possible due to economic reasons. Moreover, we were not able to interview the patients’ partners in order to get a better understanding of the actual potency rates.

Another functional and crucial outcome of RP is continence. In this study, we achieved a low urinary incontinence rate (9.9%) with a waterproof ureterovesical anastomosis test (performed with 200 mL of normal saline) and a longer average Foley catheter time (7 days) for healthier healing of the anastomosis line. In our study, the continence rate was high in both groups and there was no significant difference between the groups. It is interesting to note that none of the 9.9% of the patients that stated that they were not completely dry used pads. Zorn et al. [23] suggested that continence rates increase with increasing experience in RALRP, which is very promising.

 The overall expected benefit from minimally invasive surgery in RP can be summarized as the addition of the advantages of laparoscopic surgery to the oncologic outcomes of open RP. Biochemical Recurrence (BCR) and PSM are commonly used indicators in the assessment of oncologic outcomes after RP [24]. A PSM is determined by the stained areas of the soft tissue on the RP specimen. The incidence of PSMs is influenced by the presence of an extra prostatic extension, with a rate that ranges from 10% to 48% [25]. Some studies have shown no significant difference in PSM and/ or BCR rates between the RRP and LRP or between the RRP and RALRP groups [24-26]. In addition, the learning curve in RALRP did not have a significant effect on the pathological outcomes as reported by Islamoglu et al. [14]. In our study, the PSM rate was 35.6% in the first 45 cases (group 1) and 26.1% for 46-91st cases (group 2). These results were similar or slightly higher compared to other studies. However, extraprostatic dissemination was detected in 4 out of 16 patients with PSM from group 1 and 4 out of 12 patients with PSM from group 2. We believe that the surgical margin positivity detected here might be due to deterioration of capsule integrity during retraction with the prograsper, which was used to facilitate dissection of the prostate. The PSM rates of patients with T2 pathologies were 32 and 19% for group 1 and 2, respectively. Although this decrease was not statistically significant, we still think it is relevant clinically. Biochemical recurrence was not detected in any of the patients with a mean follow-up of 22 months. In our study, oncological results were similar before and after the learning curve stabilization, but it was noteworthy that the number of patients with PSM in group 2 was lower in both general group and pT2 subgroup than in the group 1 (35% vs 26%, 32% vs 19%). We believe that a joint investigation with our pathology specialists is needed to better understand the reason for the incompatibility of extraprostatic spread and biochemical recurrence.

 The incidence of complications after RALRP ranges from 5% to 19.6% [27-28]. In our study, 14 patients developed 15 complications. This rate was consistent with the literature and similar complication rates were observed in both groups (17.7% vs 15.5%). The majority of complications seen in both groups were minor complications (75% vs 71%).

Limitations of this study include the following: low number of patients in the groups, which decreases the sample value, evaluation of postoperative continence and potency rates with patient interviews without the use of questionnaires or tests. In addition, short-term follow-up was another limitation of the study. Therefore, more studies with larger sample sizes, longer follow-up times, and adequate tools for evaluating functional outcomes are needed.

## Conclusions

Nowadays, along with oncological outcomes, achieving a good quality of life has become more prominent in the treatment of prostate cancer. Therefore, the functional results of the surgical procedure and meeting patients’ expectations are crucial. Here we investigated the learning curve for bilateral nerve-sparing RALRP, which requires advanced surgical experience, with a surgeon that has sufficient experience in RRP and LRP. Analyses of 91 cases showed that positive operative, pathological, and functional outcomes can be obtained with a relatively short learning curve.
